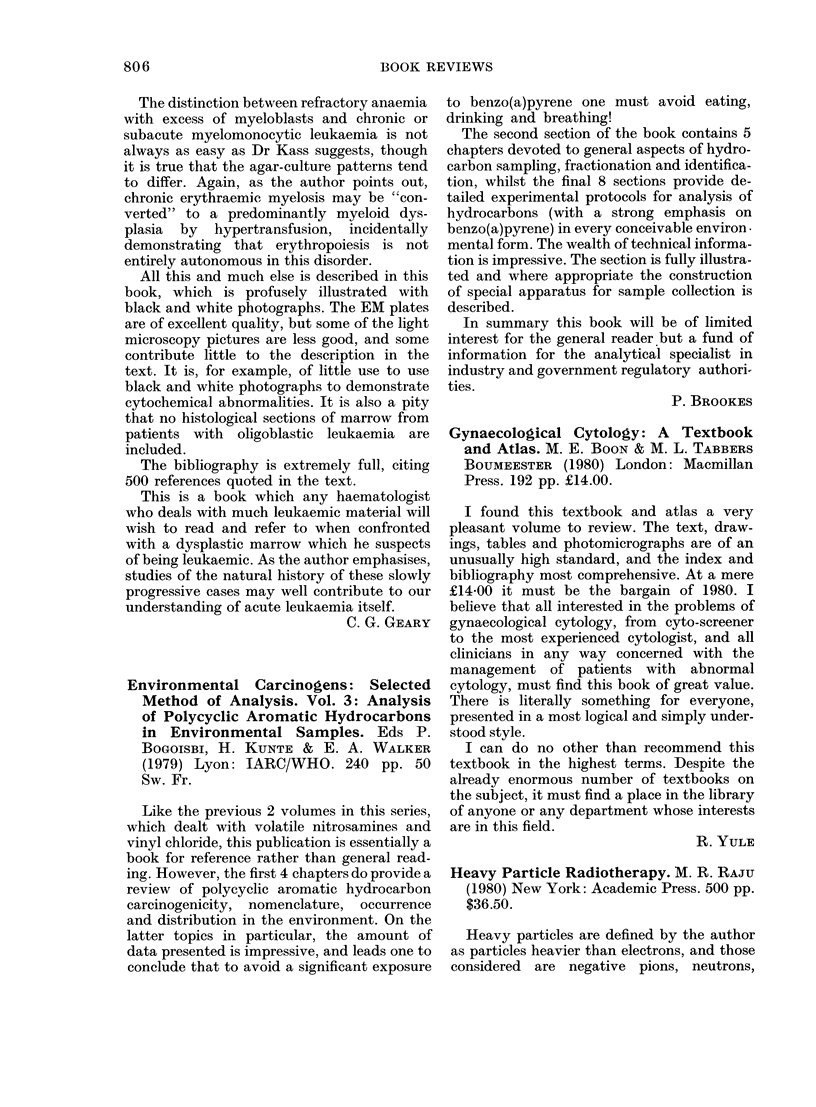# Gynaecological Cytology: A Textbook and Atlas

**Published:** 1980-11

**Authors:** R. Yule


**Gynaecological Cytology: A Textbook and Atlas.** M. E. BOON & M. L. TABBERS BOUMEESTER (1980) London: Macmillan Press. 192 pp. £14.00.

I found this textbook and atlas a very pleasant volume to review. The text, drawings, tables and photomicrographs are of an unusually high standard, and the index and bibliography most comprehensive. At a mere £14·00 it must be the bargain of 1980. I believe that all interested in the problems of gynaecological cytology, from cyto-screener to the most experienced cytologist, and all clinicians in any way concerned with the management of patients with abnormal cytology, must find this book of great value. There is literally something for everyone, presented in a most logical and simply understood style.

I can do no other than recommend this textbook in the highest terms. Despite the already enormous number of textbooks on the subject, it must find a place in the library of anyone or any department whose interests are in this field.

R. YULE